# Development of Two Diagnostic Prediction Models for Leptomeningeal Metastasis in Patients With Solid Tumors

**DOI:** 10.3389/fneur.2022.899153

**Published:** 2022-05-23

**Authors:** Tianqi Gao, Fengxi Chen, Man Li

**Affiliations:** Department of Oncology, The Second Hospital of Dalian Medical University, Dalian, China

**Keywords:** leptomeningeal metastasis (LM), prediction model, metastasic carcinoma, carcinomatous meningitis, diagnostic model

## Abstract

**Objectives:**

For accurate diagnosis of leptomeningeal metastasis (LM) and to avoid unnecessary examinations or lumber puncture (LP), we develop two diagnostic prediction models for patients with solid tumors.

**Study Design, Setting, and Participants:**

This is a retrospective cohort study launched at the Second Affiliated Hospital of Dalian Medical University. In total, 206 patients who had been admitted between January 2005 and December 2021 with a solid tumor and clinical suspicion of LM were enrolled to develop model A. In total, 152 patients of them who underwent LPs for cytology and biochemistry were enrolled to develop model B.

**Model Development:**

Diagnostic factors included skull metastasis, active brain metastasis, progressed extracranial disease, number of extracranial organs involved, number of symptoms, cerebrospinal fluid (CSF) protein, and CSF glucose. The outcome predictor was defined as the clinical diagnosis of LM. Logistic least absolute shrinkage and selection operator (LASSO) regression was used to identify relevant variables and fit the prediction model. A calibration curve and the concordance index (c-index) were used to evaluate calibration and discrimination ability. The *n*-fold cross-validation method was used to internally validate the models. The decision curve analysis (DCA) and the interventions avoided analysis (IAA) were used to evaluate the clinical application.

**Results:**

The area under the curve (AUC) values of models A and B were 0.812 (95% CI: 0.751–0.874) and 0.901 (95% CI: 0.852–0.949). Respectively, compared to the first magnetic resonance imaging (MRI) and first LP, models A and B showed a higher AUC (model A vs. first MRI: 0.812 vs. 0.743, *p* = 0.087; model B vs. first LP: 0.901 vs. 0.800, *p* = 0.010). The validated c-indexes were 0.810 (95% CI: 0.670–0.952) and 0.899 (95% CI: 0.823–0.977). The calibration curves show a good calibrated ability. The evaluation of clinical application revealed a net clinical benefit and a reduction of unnecessary interventions using the models.

**Conclusions:**

The models can help improve diagnostic accuracy when used alone or in combination with conventional work-up. They also exhibit a net clinical benefit in medical decisions and in avoiding unnecessary interventions for patients with LM. Studies focused on external validation of our models are necessary in the future.

## Introduction

Leptomeningeal metastasis (LM) refers to the dissemination of malignant cells in subarachnoid space, pia, and arachnoid mater (1), which is a devastating condition associated with metastatic solid tumors. Approximately 4–15% of all the patients with solid tumors develop LM; however, these data are mainly from autopsy and may not represent a current clinical incidence due to the development of imaging and increased patient survival through more effective treatment strategies. The survival of patients with LM is about 6–8 weeks without tumor-specific treatment and is prolonged to 1.75–6 months with LM-directed treatment (2–5). In the past few decades, the treatment regimens of LM have been greatly updated with novel targeted therapies and immunotherapy. Osimertinib targeting epidermal growth factor receptor (EGFR) mutations in patients with non-small cell lung cancer and intrathecal trastuzumab in HER2-positive breast cancer patients with LM have shown therapeutic efficacy with the median overall survival exceeding 13 months (6, 7). Immunotherapy, such as pembrolizumab, also displayed a considerable central nervous system response and a manageable toxicity profile in patients with LM (8). These data indicate that the survival of patients with LM could be improved with early diagnosis and rationale management, rather than taking it as the end of life for patients.

The diagnosis of LM is relatively complicated and difficult, which currently depends on clinical signs, cerebrospinal fluid (CSF) cytopathology, and manifestations of neuroimaging. According to the European Society for Medical Oncology and the European Association of Neuro-Oncology (EANO-ESMO) guidelines, there are four evidence levels of LM diagnosis of LM: confirmed, probable, possible, and lack of evidence (5). Confirmed diagnosis means that positive CSF cytology or biopsy is found in suspected patients. Probable diagnosis means that clinical findings and neuroimaging are simultaneously present but without pathological evidence. These two levels refer to a definite clinically diagnosis of LM. However, the estimated sensitivities of magnetic resonance imaging (MRI) and CSF cytology in large cohorts of patients with LM are only 66–98% and 50–67%, respectively. In the aspect of technical requirements, the CSF volume for the assay should be sufficient, at least 10 ml, and tested as soon as possible to avoid false-negative results (9). Even though, some patients may reject a lumber puncture (LP) because of inability to coordinate, concomitant contraindications, or refusal of invasive operations in clinical practice. Patients only with symptoms or imaging manifestations are classified as a possible level of LM. The most common symptoms of LM are as follows: headache, nausea and vomiting; diplopia, facial weakness, and changes in hearing; gait difficulties; paresthesia; neck and back pain; and mental changes, which are not specific and could exist in other situations such as infectious meningitis and side effects of anticancer treatments (10). Because of the insufficient sensitivity of CSF cytology and MRI and non–specific clinical signs, patients with a possible level of LM may be misdiagnosed or delayed.

Diagnostic prediction models are tools that combine multiple predictors by assigning relative weights to each predictor and obtain a risk or probability, which are used to estimate the probability that a specific disease or condition is present and inform patients to take further tests or initiate treatment directly (11). Here, two predictive models were developed and validated based on clinical features, symptoms, and laboratory examinations to estimate the probability of LM in suspected patients to contribute to early diagnosis and treatment.

## Methods

### Study Design, Participants, and Data Collection

**Figure 1:**
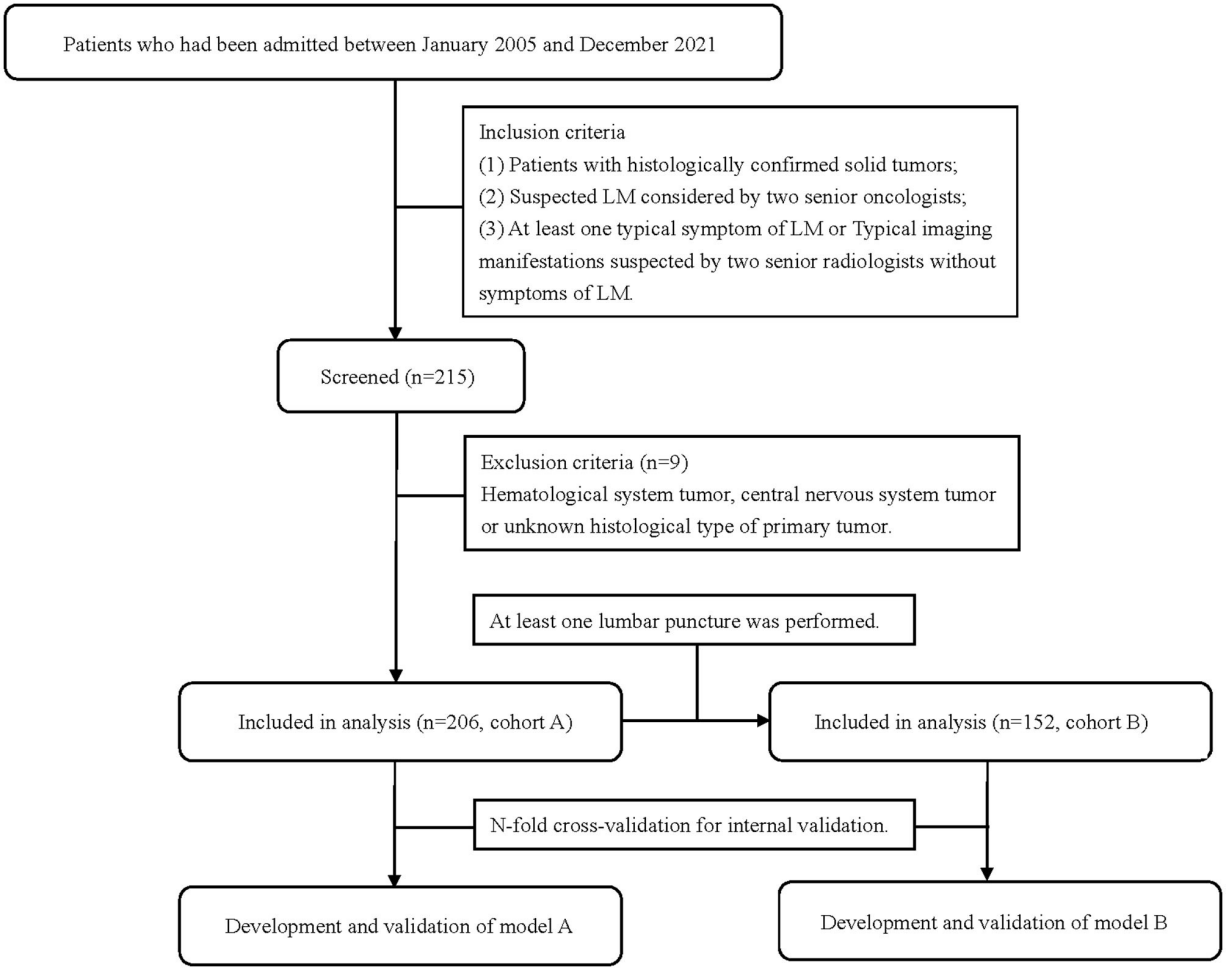
Flow chart of the study population.

This was a retrospective cohort study and was approved by the ethics committee of the Second Affiliated Hospital of Dalian Medical University (a university hospital and cancer hospital). All patients who had been admitted to the Second Affiliated Hospital of Dalian Medical University between January 2005 and December 2021 with a solid tumor and clinical suspicion of LM were included. A total of 206 patients were enrolled in cohort A to develop model A. In total, 152 patients of them who underwent lumbar punctures and obtained CSF for cytology and biochemistry were enrolled in cohort B to develop model B (Figure 1). Data were collected retrospectively through the electronic medical record system, including clinical features, test, and imaging information. Inclusion criteria were as follows: (1) patients with histologically confirmed solid tumors. (2) Suspected LM considered by two senior oncologists. (3) Exhibiting at least one typical symptom of LM including, but not limited to, headache, nausea, and vomiting; destruction of cranial nerve function; gait difficulties; paresthesia; neck and back pain and mental changes; or typical imaging manifestations suspected by two senior radiologists but without LM symptoms. Exclusion criteria were as follows: hematological system, central nervous system or unknown histological type of primary tumor. The study was designed and reported according to the transparent reporting of a multivariable prediction model for individual prognosis or diagnosis (TRIPOD) statement (11).

### Outcome and Predictors

The outcome variable and predictors were considered according to the EANO-ESMO Clinical Practice Guidelines and published literature on LM (5, 10, 12, 13). The outcome was defined as at least one of the following conditions was fulfilled: (1) positive CSF cytology on the initial lumbar puncture or repeated lumbar punctures performed within 2 weeks; (2) unequivocal evidence of LM showed on the first cerebrospinal MRI or within 2 months following the first MRI, concomitant with typical symptoms of LM. Diagnostic factors included skull metastasis, active brain metastasis, progressed extracranial disease, number of extracranial organs involved, number of symptoms, CSF protein, and CSF glucose. All predictors were evaluated and recorded when the patient was suspected of LM. Active brain metastases was defined as concomitant untreated or progressed brain metastases (14–16). Progressed extracranial disease means disease progression (limited to extracranial lesions) evaluated by clinicians according to RECIST 1.1 (17). Common symptoms of LM were classified into eight categories: headache/nausea/vomiting, cranial nerve palsies, paresthesia, gait difficulties, meningeal irritation, bowel/bladder dysfunction, neck/back/radicular pain, and seizure and mental change (5, 10, 18, 19). Symptoms were counted according to the above categories and multiple symptoms belonging to the same category were recorded as one. Values of protein and glucose were obtained from the first biochemical examination of CSF. Definitions of all predictors are summarized in the Supplementary Material. Each of the predictors was assessed blindly to each other and to the outcome.

### Model Development and Internal Validation

Statistical analyses were conducted in R Statistical Software (4.1.2) and IBM SPSS Statistics 23.0. Baseline clinical characteristics and predictors of patients in the different groups with or without LM were compared using the two-sided Chi-square and Mann–Whitney *U*-tests for categorical and continuous variables, respectively. In cohort A, the number of missing data in skull metastasis, number of extracranial organs involved, active brain metastasis, and progressed extracranial disease was 7 (3.4%), 1 (0.4%), 4 (1.9%), and 7 (3.4%), respectively. In cohort B, the number of missing data in skull metastasis, number of extracranial organs involved, active brain metastasis, progressed extracranial disease, CSF protein, and CSF glucose was 5 (3.3%), 1 (0.7%), 2 (1.3%), 5 (3.3%), 5 (3.3%), and 5 (3.3%), respectively. We used single imputation with chained equations to replace missing values in the prognostic factors and used these values in the analyses. We log-transformed the protein values in CSF because it was not normally distributed for inclusion in the imputation model. Logistic least absolute shrinkage and selection operator (LASSO) regression advocated by statisticians were used to identify relevant variables and avoid overfitting the models utilizing the “glmnet” package (20). To fit the model, the penalty term λ that is still within one standard error (SE) of the minimum binomial deviance was used for the models to select the variables for consideration of least covariates and binomial deviance at the same time. The performance of the models was assessed by using the *n*-fold cross-validation method, which was performed by randomly splitting the population into *n* = 5 exclusive and exhaustive partitions (21). Four parts were used as a training set for data imputation, variable selection, and model fitting, and one part as testing set for first-fold analysis of 5-fold cross-validations. This was repeated five times, such that each of the five data-parts was used exactly once as a testing set and the complementary 80% data were used as a training set. The mean performance of the models on the test data sets for each cross-validation was computed. The discrimination ability of the model was quantified by using the concordance index (c-index), and calibration was obtained by plotting the calibrated curve. Receiver operating characteristic (ROC) curves were plotted with the area under the curve (AUC) value to compare the diagnostic accuracy of prediction models with the first LP and the first MRI. The methodology was checked according to the PROBAST checklist to reduce the risk of bias during the development and validation process of the models (22).

### Clinical Application Analysis

Nomograms and website tools based on coefficients of the predictors were built by utilizing the “rsconnect” and “DynNom” packages. The decision curve analysis (DCA) is based on the concept of net benefit, with benefits, and harms put on the same scale so that they can be directly compared. Net benefit is similar to the idea of net profit in business and calculated as: benefit – (harm × exchange rate). For prediction models in medical practice, net benefit corresponds to each risk threshold and is defined as the observed number of true positives is corrected by the observed proportion of false positives weighted by the odds of the risk threshold, and the result is divided by the sample size. The equation is: $\frac{True\;Positives}{N}-\frac{Pt}{1-Pt}•\frac{False\;Positives}{N}$, where *N* is the total number of individuals, and Pt is the risk threshold for treatment recommendation. Similarly, the interventions avoided analysis (IAA) refers to the net reduction and is defined as the observed number of true negatives is corrected by the observed proportion of false negatives weighted by the odds of the risk threshold, and the result is divided by the sample size (23, 24). All analyses were performed in R, and the code could be found at https://github.com/agaotianqi/LMprediction.

## Results

### Patient Characteristics

The flow chart of the patient inclusion process is shown in Figure 1. In total, 206 patients were included in cohort A and 152 patients were included in cohort B. The 54 patients did not receive an LP due to the following reasons: most of them first underwent MRI first and then were clinically diagnosed with LM based on imaging evidence and clinical symptoms. They directly received further treatments and did not undergo a following LP to get a confirmed diagnosis. The decision was made by both patients and their doctors in most cases, while six of the 54 patients signed medical documents to refuse LPs even doctors suggested LPs. LPs in five patients were not successfully performed due to poor patient cooperation or doctor operation. The puncture was unsuccessful and no CSF was obtained. Three patients did not receive LPs because they were in the end-stage disease and cannot tolerate LPs. All but six patients received both brain and spine MRI. The six patients received only a brain MRI during their routine reviews and were suspected to have LM manifestations. Four of them were finally diagnosed with LM, and the other two were not because of negative cytology and no concomitant symptoms.

The basic demographic data, clinical features, and predictors of the participants are summarized in Table 1. The difference between the groups with and without LM was considered to be statistically significant when the value of *p* < 0.05. In cohort A, 143 patients were diagnosed with LM. The number of patients with confirmed and probable diagnosis of LM was 87 (42.2%) and 56 (27.1%), respectively. The median age was 54 and 56 in populations with and without LM. In cohort B, 95 patients were diagnosed with LM. The number of patients with confirmed and probable diagnosis was 87 (57.2%) and 8 (5.3%), respectively. The median age was 52 and 55 years in populations with and without LM. The percentage of LM patients with lung cancer, breast cancer, and other cancer types in cohorts A and B is 62.9%, 23.8%, 13.3%, and 63.2%, 25.3%, and 11.6%, respectively. Patients in the LM group exhibits more clinical symptoms than those in the non-LM group (*p* < 0.0001) in both cohorts, and a higher rate of active brain metastasis but no statistical significance in cohort A (cohort A, *p* = 0.058 and cohort B, *p* = 0.029). For patients in cohort B, the levels of protein and chloride in CSF were higher and the glucose in CSF was lower in the LM group than in the non-LM group (*p* < 0.0001; *p* = 0.006; *p* < 0.0001). There were no significant differences in other features, including gender, stage, cancer type, metastasis site, skull metastasis, progressed extracranial disease, and number of extracranial organs involved.

**Table 1:** Differences of clinical characteristics between patients with leptomeningeal metastasis (LM) and non-LM.

	**Cohort A**			**Cohort B**		
	**LM patients**	**Non-LM patients**	**P-value**	**LM patients**	**Non-LM patients**	**P-value**
Total patients No.	143(69.4)	63(30.6)		95(62.5)	57(37.5)	
Confirmed LM	87(42.2)	-		87(57.2)	-	
Probable LM	56(27.1)	-		8(5.3)	-	
**Gender**						
Male	45(31.5)	27(42.9)	0.1142	31(32.6)	26(45.6)	0.1095
Female	98(68.5)	36(57.1)		64(67.4)	31(54.4)	
Mean age (min-max, years)	54(24–78)	56(19–79)	0.2765	52(24–78)	55(19–79)	0.1566
**Cancer type**						
breast cancer	34(23.8)	13(20.6)	0.8191	24(25.3)	12(21.1)	0.6884
lung cancer	90(62.9)	40(63.5)		60(63.2)	36(63.2)	
others	19(13.3)	10(15.9)		11(11.6)	9(15.8)	
Stage^*^						
I	0(0)	2(3.2)	0.2263	0(0)	2(3.5)	0.2488
II	1(0.7)	1(1.6)		0(0)	1(1.8)	
III	11(7.7)	3(4.8)		7(7.4)	3(5.3)	
IV	126(88.1)	54(85.7)		84(88.4)	48(84.2)	
NA	5(3.5)	3(4.8)		4(4.2)	3(5.3)	
**Metastasis site**						
Bone	78(54.5)	30(47.6)	0.359	49(51.6)	26(45.6)	0.4764
lung	43(30.1)	22(34.9)	0.49	27(28.4)	17(29.8)	0.8535
liver	21(14.7)	9(14.3)	0.9403	15(15.8)	9(15.8)	>0.99
brain	70(49.0)	26(41.3)	0.3085	48(50.5)	23(40.4)	0.2235
Lymph node	56(39.2)	24(38.1)	0.885	35(36.8)	20(35.1)	0.8275
**Skull metastasis**						
Yes	21(14.7)	4(6.3)	0.1353	11(11.6)	3(5.3)	0.2852
No	116(81.1)	58(92.1)		80(84.2)	53(90.3)	
NA	6(4.2)	1(1.6)		4(4.2)	1(1.8)	
**Active brain metastasis**						
Yes	44(30.8)	11(17.5)	0.0576	29(30.5)	9(15.8)	**0.0296**
No	97(67.8)	50(79.4)		66(69.5)	46(80.7)	
NA	2(1.4)	3(4.8)		0(0)	2(3.5)	
**Number of involved extracranial organs**						
0	28(19.6)	18(28.6)	0.2789	22(23.2)	17(29.8)	0.3453
1	44(30.8)	16(25.4)		31(32.6)	16(28.1)	
2	42(29.4)	18(28.6)		24(25.3)	16(28.1)	
3	16(11.2)	5(7.9)		8(8.4)	4(7.0)	
4	9(6.3)	4(6.3)		8(8.4)	2(3.5)	
≥5	4(2.8)	1(1.6)		2(2.1)	1(1.8)	
NA	0(0)	1(1.6)		0(0)	1(1.8)	
**Number of symptoms**						
0	4(2.8)	2(3.2)	**<0.0001**	3(3.2)	2(3.5)	**<0.0001**
1	30(21.0)	16(25.4)		11(11.6)	10(17.5)	
2	47(32.9)	21(33.3)		34(35.8)	21(36.8)	
3	38(26.6)	19(30.2)		30(31.6)	19(33.3)	
4	13(9.1)	4(6.3)		8(8.4)	4(7.0)	
≥5	11(7.7)	1(1.6)		9(9.5)	1(1.8)	
**Progressed extracranial disease**						
Yes	46(32.2)	14(22.2)	0.1843	26(27.4)	11(19.3)	0.341
No	91(63.6)	48(76.2)		65(68.4)	45(78.9)	
NA	6(4.2)	1(1.6)		4(4.2)	1(1.8)	
**Biochemical test of CSF**						
Median protein (min-max, mg/L)	-	-	-	823.75(100–15000)	507.05(190.7–11121.2)	**<0.0001**
Median glucose (min-max, mmol/L)	-	-	-	2.45(0.5–5.3)	3.30(1.5–6)	**<0.0001**
Median chloride (min-max, mmol/L)	-	-	-	119.80(106.3–148.9)	121.16(110.4–129)	**0.0058**

*Values are numbers (percentages) of patients unless stated otherwise*.

* *Stage was evaluated when LM was suspected. Pathological stages were recorded in postoperative patients without metastasis. The P-values which were less than 0.05 were shown in bold*.

### Development and Internal Validation of Model A

**Figure 2:**
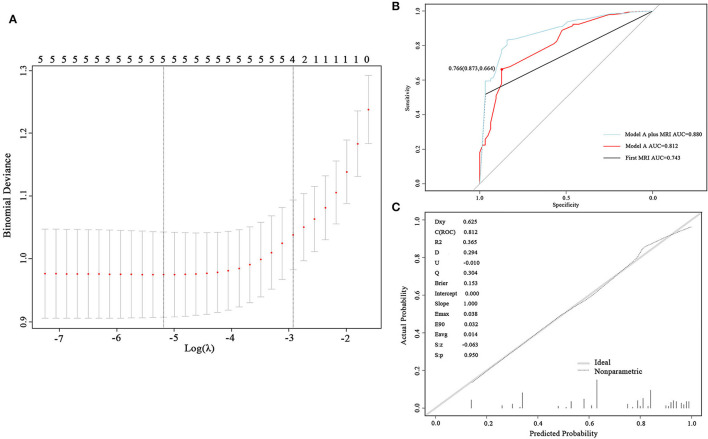
Development and validation of model A. **(A)** Cross-validation plot for the penalty term. Two solid lines corresponded to five and four variables when λ was minimum and minimum within one SE, respectively. **(B)** ROC curves of model A and the first magnetic resonance imaging (MRI). The AUC was 0.812 for model A and 0.743 for the first MRI. **(C)** The calibrated curve of model A. The gray solid line represents the actual probability, and the dotted line represents the prediction probability of model A. AUC, area under the curve; ROC, receiver operating characteristic.

Variables, including skull metastasis, active brain metastasis, progressed extracranial disease, number of extracranial organs involved, and number of symptoms were selected as probable predictors in models A and B. Additional two predictors, protein and glucose in CSF, were added to the list of model B because these patients had taken at least one LP in their diagnostic procedures. LASSO regression was used to find the optimal model *via* cross-validation and further shrinkage by increasing λ. To develop model A, five predictors were all involved in the model when λ was minimum (λ = 0.0056) and four predictors remained when λ was increased within one SE of the minimum (λ = 0.0536) (Figure 2A). The latter λ was selected to avoid overfitting, and the four predictors were skull metastasis, active brain metastasis, progressed extracranial disease, and number of symptoms, which were used to fit model A. The coefficients of each predictor are shown in Table 2. The ROC curve and calibrated curve demonstrate that model A had effective discrimination ability (AUC = 0.812, 95% CI: 0.751–0.874) and calibration ability (Figures 2B,C). The sensitivity and specificity at the cutoff value were 87.3% and 66.4%, and the corresponding positive predictive value and negative predictive value was 100% and 66.7%. The performance of model A was also evaluated in subgroups of confirmed and probable patients, and the AUC value was 0.840 (95% CI: 0.777–0.902) and 0.771 (95% CI: 0.687–0.855), respectively (Supplementary Material). Compared with the first MRI patients, the ROC curve of model A showed a higher, but not statistically significant AUC value (AUC = 0.743, 95% CI: 0.696–0.789; *p* = 0.087). Then, we combined model B with the first MRI to make a joint diagnosis, and found that the AUC value increased significantly (AUC = 0.880, 95% CI: 0.696–0.789; *p* < 0.001) (Figure 2B; Table 3). Next, the 5-fold cross-validation analysis was carried out to check how well the model A generalizes to a new data. The mean c-index of model A on the test data sets was 0.810 (95% CI: 0.670–0.952), and the Brier score was 0.151, indicating that model A performed well to predict LM in suspected patients.

**Table 2:** Coefficients of predictors in model A.

**Predictors**	**Estimate**	**Std. Error**	**z-value**	**p-vaule**
Skull metastasis	1.0949	0.6480	1.6900	0.0911
Active brain metastasis	0.7830	0.4324	1.8110	0.0702
Number of symptoms	1.1753	0.2008	5.8540	<0.0001
Progressed extracranial disease	0.9627	0.4160	2.3140	0.0207

**Table 3:** Diagnostic accuracy of magnetic resonance imaging (MRI).

	**Neuroimaging findings in LM patients**	**No neuroimaging findings** **in non-LM patients**
First MRI	74	61
Second MRI	10	0
Total patients	143	63

*First MRI, Second MRI, The number of patients got characteristic findings from the first and the second MRI in patients with LM and the number of patients got no characteristic finding from the first and the second MRI in patients with non-LM; Total patients, The number of total patients diagnosed with LM and without LM; LP, lumber puncture*.

### Development and Internal Validation of Model B

**Figure 3:**
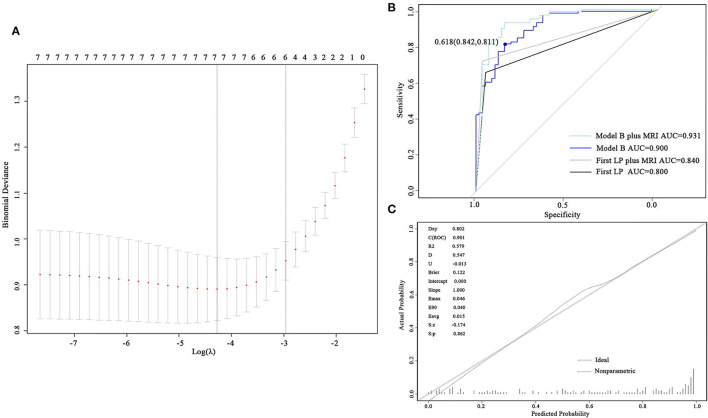
Development and validation of model B. **(A)** Cross-validation plot for the penalty term. Two solid lines corresponded to seven and six variables when λ was minimum and minimum within one SE, respectively. **(B)** ROC curves of model B and the first LP. The AUC was 0.901 for model B and 0.800 for the first lumber puncture. **(C)** The calibrated curve for model B. The gray solid line represents the actual probability, and the dotted line represents the prediction probability of model B. AUC, area under the curve; ROC, receiver operating characteristic; LP, lumber puncture.

To develop model B, the values of protein in CSF were log-transformed to better fit the model to a normal distribution. Seven and six predictors were involved when selecting the minimum λ (λ = 0.0139) and the minimum within one SE (λ = 0.0518), respectively (Figure 3A). The six predictors constituting model B were active brain metastasis, progressed extracranial disease, number of symptoms, number of extracranial organs involved, CSF protein, and CSF glucose. The coefficients of the predictors are shown in Table 4. The ROC curve (AUC = 0.901, 95% CI: 0.852–0.949) and the calibrated curve of model B are shown in Figures 3B,C. The sensitivity and specificity at the cutoff value were 84.2% and 81.1%, and the corresponding positive predictive value and negative predictive value was 100% and 69.2%. The AUC value of model B in patients with confirmed LM was up to 0.928 (95% CI: 0.888–0.969) (Supplementary Material). Compared to the first LP patients received, the ROC curve of model B showed a better AUC value and discrimination ability than the first LP cytology (AUC = 0.800, 95% CI: 0.744–0.856; *p* = 0.010) (Figure 3B; Table 5). Because MRI will also be used in clinical practice, we performed the ROC curve of MRI plus LP and the AUC was 0.840 (95% CI: 0.789–0.892). Compared with the diagnostic accuracy of model B, there was no significant difference (*p* = 0.118). However, we suggest that model B can also be combined with MRI for LM diagnosis. Then, we performed the ROC curve of MRI plus model B and found that the AUC value rose to 0.931 (95% CI: 0.886–0.976), which was significantly higher than LP plus MRI (*p* = 0.002) (Figure 3B). Internal validation was also done on model B by the 5-fold cross-validation analysis. The mean c-index of model B was 0.899 (95% CI: 0.823–0.977), and the Brier score was 0.118 in the test data sets. Collectively, model B also had good discrimination and calibration performance in both the original cohort and cross-validation test data sets.

**Table 4:** Coefficients of predictors in model B.

**Predictors**	**Estimate**	**Std. Error**	**z-value**	**p-vaule**
Progressed extracranial disease	0.8187	0.6210	1.3180	0.1874
Active brain metastasis	1.5489	0.6916	2.2390	0.0251
Number of involved extracranial organs	0.2537	0.2209	1.1480	0.2508
Number of symptoms	1.3590	0.2862	4.7480	<0.0001
log_CSF pro^*^	1.3112	0.6692	1.9590	0.0264
CSF glu	−1.0052	0.2589	−3.8830	0.0001

* *log transformed value of protein in cerebrospinal fluid (CSF)*.

**Table 5:** Diagnostic accuracy of LP.

**LP**	**Cytology**	**LM group**	**non-LM group**	**Total**
The first LP	Positive	62	0	62
	Equivocal	12	3	15
	Negative	21	54	75
After the second LP	Positive	82	0	82
	Equivocal	5	0	5
	Negative	8	57	65
After the third or later LP	Positive	87	0	87
	Equivocal	0	0	0
	Negative	8	57	65
Total	95	57	152

*LP, lumber puncture*.

### Clinical Application Evaluation

**Figure 4:**
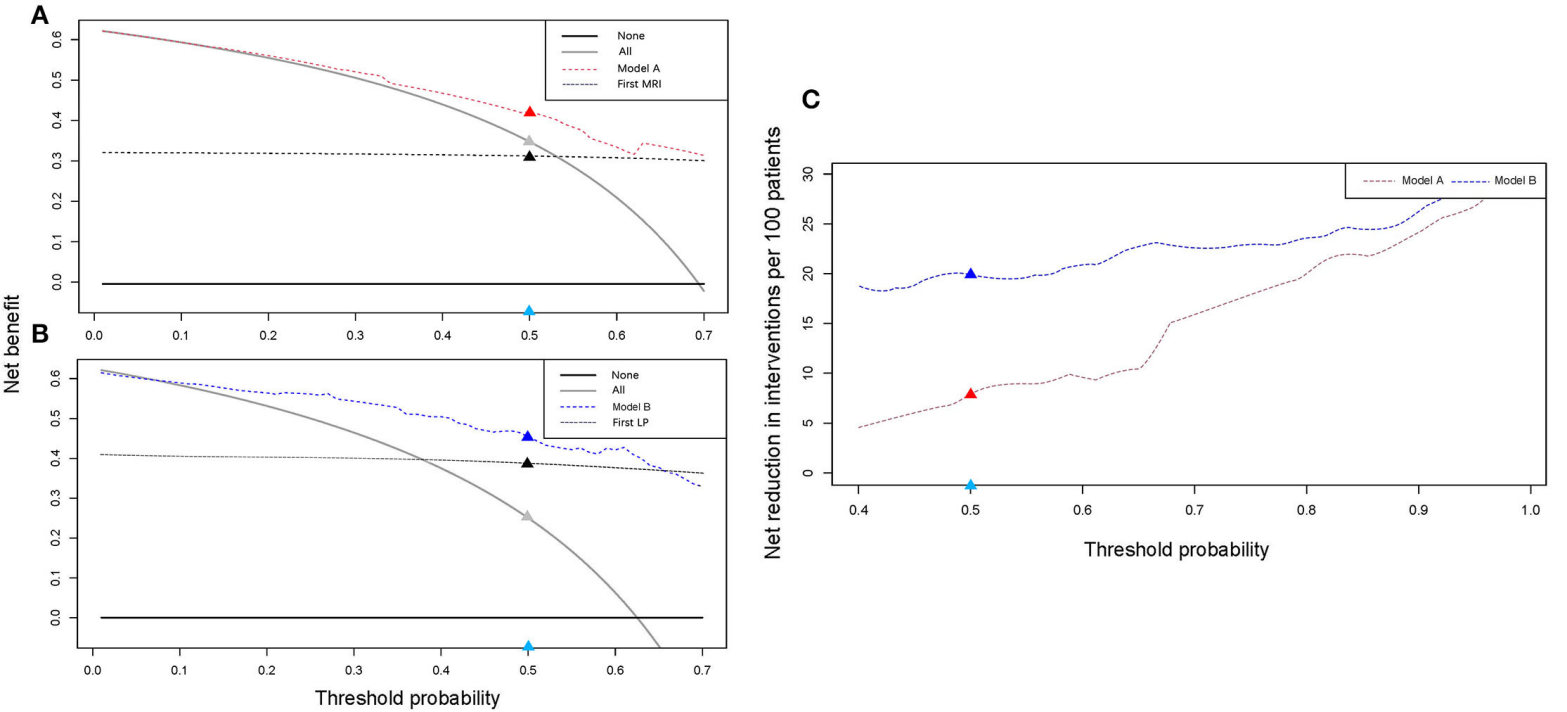
Clinical application evaluation. DCA and IAA for models A and B reveal a net benefit in the clinical decision compared to “treat all” or “treat none” and net reduction in unnecessary intervention. **(A,B)** DCA curves for models A and B. Compared with the first MRI, model A exhibits a net benefit when the threshold was <0.7. Compared with the first LP, model B exhibits a net benefit when the threshold was <0.66. **(C)** IAA curves for models A and B. Models A and B can help avoid unnecessary interventions. For example, in **(A)**, when the threshold was set at 0.5 (the light blue dot), the corresponding net benefit by using model A (the red dot) was higher than that of “treat all” (the gray dot) and the first MRI (the black dot). In **(C)**, the net reduction in interventions was increased by using the models (the red and blue dots). DCA, decision curve analysis; IAA, interventions avoided analysis; LP, lumber puncture.

To evaluate the clinical application of the models, DCA and IAA were performed. As shown in Figures 4A,B, the net benefit of treating selected patients using predictive models was improved compared to all patients (“treat all”) or no patients (“treat none”). For example, at the threshold probability of 50%, the net benefit is equal to 8 or 20 of 100 patients benefiting from treatment by using model A or B. We also compared models A and B with the result of the first MRI and the first LP for cytological results and found that the net benefit was improved when the threshold probability was set at < 70% and 66%, respectively. IAA illustrated that unnecessary interventions, which can be redundant examinations or LPs (Figure 4C), were reduced. The net reduction was equal to further diagnostic MRI or LPs in eight of 100 patients using model A, and a second LP was avoided in 20 of 100 patients using model B.

### Building Visual Predictive Models

**Figure 5:**
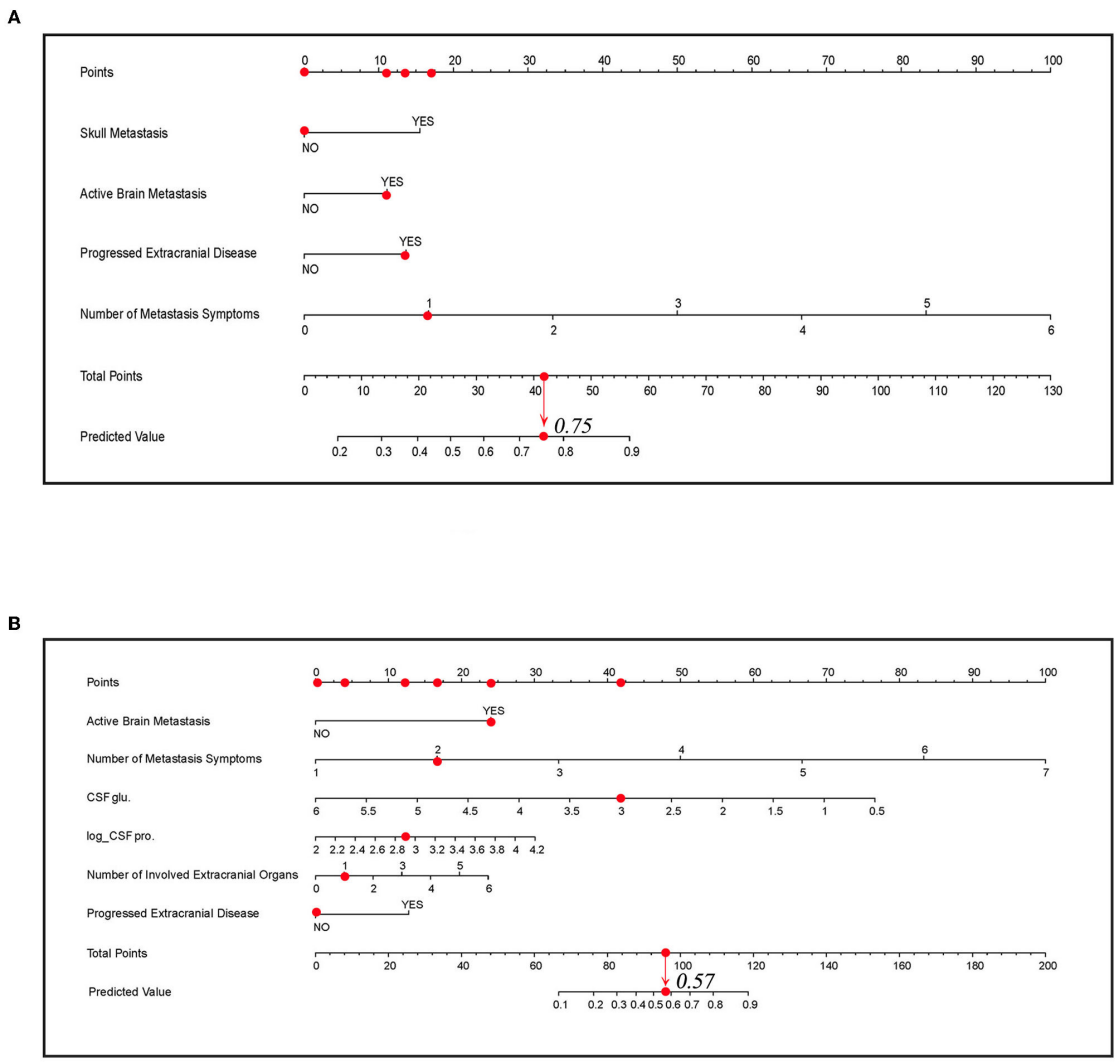
The nomogram for predicting LM. **(A)** The nomogram of model A. **(B)** The nomogram of model B. The scores for each predictor correspond to the uppermost point axis (the red dots on the top line). The total point line is at the bottom, and each predictor point was summed to get the total points (the red dot on the total point line). Then, the prediction value was obtained. For example, in **(B)**, when a patient presents active brain metastasis, two LM symptoms, one involved extracranial organ, and the value of glucose and log-transformed value of proteins in cerebrospinal fluid (CSF) is 3 and 2.9, he will get a point of 87 and the corresponding prediction value is 0.57. glu, glucose; pro., protein; Log_CSF pro, log transformed value of protein in CSF.

Two nomogram prediction tools were built, and two web tools were developed to visualize the models based on the predictor coefficients of each model. Nomograms A and B are shown in Figures 5A,B. For patients who had laboratory examination results of CSF, nomogram B and website B should be selected for prediction because of their applicability and relatively better performance. For binary variables, “1” means “YES” and “0” means “NO” in the calculators of the websites. The web tools are as follows: https://lmpredictors.shinyapps.io/dynnomapp/ (website A); https://lmpredictor.shinyapps.io/dynnomapp/ (website B).

## Discussion

In recent decades, the incidence of LM has increased due to increased patient survival through better tolerated and more effective treatment strategies, which makes LM a cause of considerable morbidity and mortality. Treatment strategies, including systemic therapy, radiation, and intrathecal therapy, were considered as positive prognostic factors of patients with LM and have shown evidence of survival benefits in retrospective and prospective cohort studies (25–29). Recent advances in targeted therapy and immunotherapy, which could overcome the obstruction effect of blood–brain barrier, have significantly impacted the prognosis of patients with LM (6, 30, 31). Therefore, early and accurate diagnosis of LM is of great importance to initiate treatment without delay, prevent progressive neurological symptoms, and eventually prolong survival.

Despite its widespread use in clinical practice, there were obvious limitations of CSF cytology and cerebrospinal MRI. CSF cytology depends on the quality of the samples and the subjective experience of pathologists. Normally, more than one LP is required to establish the diagnosis (31), which may increase the risk of infection, bleeding, or cerebral hernia. In our study, the first LP in patients with LM had a sensitivity of 65.3% and 93.0% (Table 5), which was similar to previous research (32–35). MRI may present a false-negative result at the early stage of LM, or present abnormal enhancement similar to LM in case of infection, intracranial hypotension, and recent radiation or surgery. Compared to the conventional work-ups, liquid biopsy seems to be a promising diagnostic technology. Isolation and quantification of circulating tumor cells in CSF by using flow cytometry based on the expression of epithelial cell adhesion molecules has shown a better sensitivity of 76–100% [36]. However, the standardization of detection methodology is needed to ensure reproducible and reliable results across institutions. The relatively expensive cost of the technology also limits its clinical applications. An additional emerging technology, cell-free tumor DNA (ctDNA) detection in CSF, can reveal LM-specific genomic alternations. It is more extensively utilized in finding resistance mutations or driver mutations to guide treatment than for diagnosis [36].

To overcome the difficulties mentioned above, we sought to provide a more accurate and economic diagnostic model by adding diagnostic variables, including clinical features, symptoms, and examinations. According to whether patients underwent a lumbar puncture, two diagnostic prediction models were built for patients with suspected LM considered by clinicians. LMs often occur in advanced patients with a largely intracranial and extracranial tumor burden and are mainly from direct invasion of brain parenchymal metastases. Some cancer cells also entered the CSF through cranial and spinal nerves or vessels [37]. Considering the process, we thus selected skull metastasis, active brain metastasis, progressive extracranial disease, and the number of involved extracranial organs as partial predictors. Over 95% of patients with LM present abnormal CSF glucose and protein profile (19) and thus the biochemical parameters of CSF were also selected.

Both models exhibit good discrimination and calibration ability. Compared to the first MRI and the first LP, the prediction models had better AUC values (model A vs. first MRI: 0.812 vs. 0.743, *p* = 0.087; model B vs. first LP: 0.901 vs. 0.800, *p* = 0.010). We also performed a subgroup analysis in patients with confirmed and probable LM, and the AUC values were >0.7 in both models and subgroups, representing a good discrimination ability. The model seems to perform better in confirmed patients, which is not surprised because positive cytology often represents a huge tumor burden. These patients may present more symptoms and concomitant metastases, leading to a higher predictive value. However, the number of patients became smaller when they were divided into subgroups, so the performance needs to be validated in further studies. Then, we combined the models with the first MRI, and found that diagnostic accuracy increased significantly (model A plus first MRI vs. first MRI: 0.880 vs. 0.743, *p* < 0.001 and model B plus first MRI vs. first LP plus MRI: 0.931 vs. 0.840, *p* = 0.002). DCA and IAA reveal a net clinical benefit in using the prediction models and a net reduction in unnecessary interventions. Taken the results together, the models can be included in the LM diagnosis procedure. For example, in the case of a patient with clinical suspicion, doctors could first apply model A to judge the probability of LM and make decisions to initiate treatment or further MRI and LPs because of its net benefit in decisions and net reduction in unnecessary examinations. If MRI was done, it was suggested to combine model A with MRI for a joint diagnosis, as the diagnostic accuracy was significantly higher. If an LP was performed, model B could be used alone or in combination with MRI to decide on further treatment and avoid a needless second LP. For the convenience of clinicians, two visual nomograms were built and two website tools were developed to make the calculation easier and more accurate.

The diagnosis of LM is similar to a staged process. First, we judge patients who are prone to develop LM, and then we note whether they have typical symptoms or imaging manifestations, so as to give them a possible level of diagnostics. Next, we try to upgrade the diagnostic level of these suspected patients to a clinical diagnosis and, if possible, to a confirmed level. Limited by our study design, we did not address how reliable model A or B is for classifying LM possible cases. However, it is important and deserves further study because it helps us to recognize patients who may have LM much earlier. In that case, inclusion criteria should be properly set to decide whether to select common patients or to include patients with certain clinical characteristics. Predictors need to be focused on more basic features, such as cancer type or metastasis site, because it is more like a screening process in high-risk patients. Furthermore, the outcome variable should be cautiously defined and other causes of suspected symptoms and imaging features need to be distinguished.

Of course, our study has several limitations. First, we should note that our models are not validated by external validation, which means that the estimated performance of the models is likely overstated. Before they are actually used clinically, further research focused on the external validation of the models is necessary. Additionally, validation data through other centers could provide more information to set proper thresholds for the models according to the results of the clinical application analysis. Second, the sample size of our study was small and there was a risk of selection bias like other retrospective studies.

In conclusion, we developed two diagnostic prediction models for LM in patients with solid tumors and both of the models exhibited good performance and applicable value in clinical practice. The models could improve diagnostic accuracy when were used alone or combined with conventional diagnosis methods. And they exhibit advantages in clinical benefit in medical decisions and avoiding unnecessary examinations in patients with LM.

## Data Availability Statement

The raw data supporting the conclusions of this article will be made available by the authors, without undue reservation.

## Ethics Statement

The studies involving human participants were reviewed and approved by the Ethics Committee of the Second Hospital of Dalian Medical University. The patients/participants provided their written informed consent to participate in this study. Written informed consent was obtained from the individual(s) for the publication of any potentially identifiable images or data included in this article.

## Author Contributions

TG designed the study, collected the data, developed the model, and completed the manuscript. FC collected the data and developed the model. ML supervised the study and reviewed this manuscript. All authors contributed to the article and approved the submitted version.

## Conflict of Interest

The authors declare that the research was conducted in the absence of any commercial or financial relationships that could be construed as a potential conflict of interest.

## Publisher's Note

All claims expressed in this article are solely those of the authors and do not necessarily represent those of their affiliated organizations, or those of the publisher, the editors and the reviewers. Any product that may be evaluated in this article, or claim that may be made by its manufacturer, is not guaranteed or endorsed by the publisher.
