# Supplementary material for: Development of Two Diagnostic Prediction Models for Leptomeningeal Metastasis in Patients With Solid Tumors
Source: Front Neurol. 2022 May 23;13:899153. doi: 10.3389/fneur.2022.899153 (PMC9168081; doi:10.3389/fneur.2022.899153)
Supplement: Supplementary file 1 [file Table_1.DOCX]

Supplementary file：Definition and statistical method of predictors

| Predictors | Definition | Type of variable | Recording | Statistical analysis |
| --- | --- | --- | --- | --- |
| skull metastasis | Evidence of skull bone metastasis revealed by CT, MRI, systemic bone imaging, or pathological biopsy | categorical  (nominal) | Yes/NO | Yes=1; No=0 |
| active brain metastasis | Concomitant untreated or progressed brain metastasis | categorical  (nominal) | Yes/NO | Yes=1; No=0 |
| progressed extracranial disease | Disease progression evaluated by clinicians according to RECIST 1.1 (limited to extracranial lesions) | categorical  (nominal) | Yes/NO | Yes=1; No=0 |
| number of involved extracranial organs | The number of all extracranial organs involved by tumor metastases | numerical  (discrete) | numerical value | recorded value |
| number of symptoms | The number of symptoms counted according to the categories (multiple symptoms belonging to the same category were recorded as one) | numerical  (discrete) | numerical value | recorded value |
| protein in CSF | Protein concentration of the first CSF biochemical examination | numerical  (continuous) | test value  (mg/L) | recorded value |
| glucose in CSF | Glucose concentration of the first CSF biochemical examination | numerical  (continuous) | test value  (mg/L) | recorded value |
| Eight categories of symptoms: Headache/Nausea/vomiting, Cranial nerve palsies, Paresthesia, Gait difficulties, Meningeal irritation, Bowel/bladder dysfunction, Neck/back/radicular pain, Seizure and Mental changes. | | | | |
