# Supplementary material for: Development of Two Diagnostic Prediction Models for Leptomeningeal Metastasis in Patients With Solid Tumors
Source: Front Neurol. 2022 May 23;13:899153. doi: 10.3389/fneur.2022.899153 (PMC9168081; doi:10.3389/fneur.2022.899153)
Supplement: Supplementary file 2 [file Table_2.DOCX]

| **Signaling questions** | **Answers** | | |
| --- | --- | --- | --- |
| **Domain 1: Participants** | | | |
| Background  The overall aim for prediction models is to generate absolute risk predictions that are correct in new individuals. Certain data sources or designs are not suited to generate absolute probabilities. Problems may also arise if a study inappropriately includes or excludes participant groups from entering the study. | | | |
| **1.1 Were appropriate data sources used, e.g., cohort, RCT, or nested case–control study data?**  Yes/probably yes: If a cohort design (including RCT or proper registry data) or a nested case-control or case-cohort design (with proper adjustment of the baseline risk/hazard in the analysis) has been used.  No/probably no: If a non-nested case-control design has been used.  No information: If the method of participant sampling is unclear. | Yes/  probably yes  □ | No/  probably no  🗹 | No  Information  □ |
| **1.2 Were all inclusions and exclusions of participants appropriate?**  Yes/probably yes: If inclusion and exclusion of participants was appropriate, so participants correspond to unselected participants of interest.  No/probably no: If participants are included who would already have been identified as having the outcome and so are no longer participants at suspicion of disease (diagnostic studies) or at risk of developing outcome (prognostic studies), or if specific subgroups are excluded that may have altered the performance of the prediction model for the intended target population.  No information: When there is no information on whether inappropriate inclusions or exclusions took place. | Yes/  probably yes  🗹 | No/  probably no  □ | No  Information  □ |
| **Risk of bias introduced by participants or data sources**  Low risk of bias: If the answer to all signaling questions is “Yes” or “Probably yes,” then risk of bias can be considered low. If ≥1 of the answers is “No” or “Probably no,” the judgment could still be “Low risk of bias” but specific reasons should be provided why the risk of bias can be considered low.  High risk of bias: If the answer to any of the signaling questions is “No” or “Probably no,” there is a potential for bias, except if defined at low risk of bias above.  Unclear risk of bias: If relevant information is missing for some of the signaling questions and none of the signaling questions is judged to put this domain at high risk of bias. | Low risk of bias  □ | High risk of bias  🗹 | Unclear risk of bias  □ |
| **Domain 2: Predictors** | | | |
| Background  Bias in model performance can occur when the definition and measurement of predictors is flawed. Predictors are the variables evaluated for their association with the outcome of interest. Bias can occur, for example, when predictors are not defined in a similar way for all participants or knowledge of the outcome influences predictor assessments. | | | |
| **2.1 Were predictors defined and assessed in a similar way for all participants?**  Yes/probably yes: If definitions of predictors and their assessment were similar for all participants.  No/probably no: If different definitions were used for the same predictor or if predictors requiring subjective interpretation were assessed by differently experienced assessors.  No information: If there is no information on how predictors were defined or assessed. | Yes/  probably yes  🗹 | No/  probably no  □ | No  Information  □ |
| **2.2 Were predictor assessments made without knowledge of outcome data?**  Yes/probably yes: If outcome information was stated as not used during predictor assessment or was clearly not (yet) available to those assessing predictors.  No/probably no: If it is clear that outcome information was used when assessing predictors.  No information: No information on whether predictors were assessed without knowledge of outcome information. | Yes/  probably yes  🗹 | No/  probably no  □ | No  Information  □ |
| **2.3 Are all predictors available at the time the model is intended to be used?**  Yes/probably yes: All included predictors would be available at the time the model is intended to be used for prediction.  No/probably no: Predictors would not be available at the time the model is intended to be used for prediction.  No information: No information on whether predictors would be available at the time the model is intended to be used for prediction. | Yes/  probably yes  🗹 | No/  probably no  □ | No  Information  □ |
| **Risk of bias introduced by predictors or their assessment**  Low risk of bias: If the answer to all signaling questions is “Yes” or “Probably Yes,” then risk of bias can be considered low. If ≥1 of the answers is “No” or “Probably no,” the judgment could still be “Low risk of bias” but specific reasons should be provided why the risk of bias can be considered low, e.g., use of objective predictors not requiring subjective interpretation.  High risk of bias: If the answer to any of the signaling questions is “No” or “Probably no,” there is a potential for bias.  Unclear risk of bias: If relevant information is missing for some of the signaling questions and none of the signaling questions is judged to put the domain at high risk of bias. | Low risk of bias  🗹 | High risk of bias  □ | Unclear risk of bias  □ |
| **Domain 3: Outcome** | | | |
| Background  Bias in model performance can occur when methods used to determine outcomes incorrectly classify participants with or without the outcome. Bias in methods of outcome determination can result from use of suboptimal methods, tests, or criteria that lead to unacceptably high levels of errors in outcome determination, when methods are inconsistently applied across participants, or when knowledge of predictors influence outcome determination. Incorrect timing of outcome determination can also result in bias. | | | |
| **3.1 Was the outcome determined appropriately?**  Yes/probably yes: If a method of outcome determination has been used which is considered optimal or acceptable by guidelines or previous publications on the topic.  Note: This is about level of measurement error within the method of determining the outcome (see concerns for applicability about whether the definition of the outcome method is appropriate).  No/probably no: If a clearly suboptimal method has been used that causes unacceptable error in determining outcome status in participants.  No information: No information on how outcome was determined. | Yes/  probably yes  🗹 | No/  probably no  □ | No  Information  □ |
| **3.2 Was a prespecified or standard outcome definition used?**  Yes/probably yes: If the method of outcome determination is objective, or if a standard outcome definition is used, or if prespecified categories are used to group outcomes.  No/probably no: If the outcome definition was not standard and not prespecified.  No information: No information on whether the outcome definition was prespecified or standard. | Yes/  probably yes  🗹 | No/  probably no  □ | No  Information  □ |
| **3.3 Were predictors excluded from the outcome definition?**  Yes/probably yes: If none of the predictors are included in the outcome definition.  No/probably no: If ≥1 of the predictors forms part of the outcome definition.  No information: No information on whether predictors are excluded from the outcome definition. | Yes/  probably yes  🗹 | No/  probably no  □ | No  Information  □ |
| **3.4 Was the outcome defined and determined in a similar way for all participants?**  Yes/probably yes: If outcomes were defined and determined in a similar way for all participants.  No/probably no: If outcomes were clearly defined and determined in a different way for some participants.  No information: No information on whether outcomes were defined or determined in a similar way for all participants. | Yes/  probably yes  🗹 | No/  probably no  □ | No  Information  □ |
| **3.5 Was the outcome determined without knowledge of predictor information?**  Yes/probably yes: If predictor information was not known when determining the outcome status, or outcome status determination is clearly reported as determined without knowledge of predictor information.  No/probably no: If it is clear that predictor information was used when determining the outcome status.  No information: No information on whether outcome was determined without knowledge of predictor information. | Yes/  probably yes  🗹 | No/  probably no  □ | No  Information  □ |
| **3.6 Was the time interval between predictor assessment and outcome determination appropriate?**  Yes/probably yes: If the time interval between predictor assessment and outcome determination was appropriate to enable the correct type and representative number of relevant outcomes to be recorded, or if no information on the time interval is required to allow a representative number of the relevant outcome occur or if predictor assessment and outcome determination were from information taken within an appropriate time interval.  No/probably no: If the time interval between predictor assessment and outcome determination is too short or too long to enable the correct type and representative number of relevant outcomes to be recorded.  No information: If no information was provided on the time interval between predictor assessment and outcome determination. | Yes/  probably yes  🗹 | No/  probably no  □ | No  Information  □ |
| **Risk of bias introduced by predictors or their assessment**  Low risk of bias: If the answer to all signaling questions is “Yes” or “Probably yes,” then risk of bias can be considered low.  If ≥1 of the answers is “No” or “Probably no,” the judgment could still be low risk of bias, but specific reasons should be provided why the risk of bias can be considered low, e.g., when the outcome was determined with knowledge of predictor information but the outcome assessment did not require much interpretation by the assessor (e.g., death regardless of cause).  High risk of bias: If the answer to any of the signaling questions is “No” or “Probably no,” there is a potential for bias.  Unclear risk of bias: If relevant information about the outcome is missing for some of the signaling questions and none of the signaling questions is judged to put this domain at high risk of bias. | Low risk of bias  🗹 | High risk of bias  □ | Unclear risk of bias  □ |
| **Domain 4: Analysis** | | | |
| Background  Statistical analysis is a critical part of prediction model development and validation. The use of inappropriate statistical analysis methods increases the potential for bias in reported model performance measures. Model development studies include many steps where flawed methods can distort results. We recommend reviewers seek statistical advice when completing assessments of the analysis domain. | | | |
| **4.1 Were there a reasonable number of participants with the outcome?**  Yes/probably yes: For model development studies, if the number of participants with the outcome relative to the number of candidate predictor parameters is ≥20 (EPV ≥20).*  For model validation studies, if the number of participants with the outcome is ≥100.  No/probably no: For model development studies, if the number of participants with the outcome relative to the number of candidate predictor parameters is <10 (EPV <10).*  For model validation studies, if the number of participants with the outcome is <100.  No information: For model development studies, no information on the number of candidate predictor parameters or number of participants with the outcome, such that the EPV cannot be calculated.  For model validation studies, no information on the number of participants with the outcome. | Yes/  probably yes  □ | No/  probably no  🗹 | No  Information  □ |
| **4.2 Were continuous and categorical predictors handled appropriately?**  Yes/probably yes: If continuous predictors are not converted into ≥2 categories when included in the model (i.e., dichotomized or categorized), or if continuous predictors are examined for nonlinearity using, for  example, fractional polynomials or restricted cubic splines, or if categorical predictor groups are defined using a prespecified method.  For model validation studies, if continuous predictors are included using the same definitions or transformations, and categorical variables are categorized using the same cut points, as compared with the development study.  No/probably no: If categorical predictor group definitions do not use a prespecified method.  For model development studies, if continuous predictors are converted into ≥2 categories when included in the model.  For model validation studies, if continuous predictors are included using different definitions or transformations, or categorical variables are categorized using different cut points, as compared with the development study.  No information: No information on whether continuous predictors are examined for nonlinearity and no information on how categorical predictor groups are defined.  For model validation studies, no information on whether the same definitions or transformations and the same cut points are used, as compared with the development study. | Yes/  probably yes  🗹 | No/  probably no  □ | No  Information  □ |
| **4.3 Were all enrolled participants included in the analysis?**  Yes/probably yes: If all participants enrolled in the study are included in the data analysis.  No/probably no: If some or a subgroup of participants are inappropriately excluded from the analysis.  No information: No information on whether all enrolled participants are included in the analysis. | Yes/  probably yes  🗹 | No/  probably no  □ | No  Information  □ |
| **4.4 Were participants with missing data handled appropriately?**  Yes/probably yes: If there are no missing values of predictors or outcomes and the study explicitly reports that participants are not excluded on the basis of missing data, or if missing values are handled using multiple imputation.  No/probably no: If participants with missing data are omitted from the analysis, or if the method of handling missing data is clearly flawed, e.g., missing indicator method or inappropriate use of last value carried forward, or if the study had no explicit mention of methods to handle missing data.  No information: If there is insufficient information to determine if the method of handling missing data is appropriate. | Yes/  probably yes  🗹 | No/  probably no  □ | No  Information  □ |
| **4.5 Was selection of predictors based on univariable analysis avoided?†**  Yes/probably yes: If the predictors are not selected on the basis of univariable analysis prior to multivariable modeling.  No/probably no: If the predictors are selected on the basis of univariable analysis prior to multivariable modeling.  No information: If there is no information to indicate that univariable selection is avoided. | Yes/  probably yes  🗹 | No/  probably no  □ | No  Information  □ |
| **4.6 Were complexities in the data (e.g., censoring, competing risks, sampling of control participants) accounted for appropriately?**  Yes/probably yes: If any complexities in the data are accounted for appropriately, or if it is clear that any potential data complexities have been identified appropriately as unimportant.  No/probably no: If complexities in the data that could affect model performance are ignored.  No information: No information is provided on whether complexities in the data are present or accounted for appropriately if present. | Yes/  probably yes  🗹 | No/  probably no  □ | No  Information  □ |
| **4.7 Were relevant model performance measures evaluated appropriately?**  Yes/probably yes: If both calibration and discrimination are evaluated appropriately (including relevant measures tailored for models predicting survival outcomes).  No/probably no: If both calibration and discrimination are not evaluated, or if only goodness-of-fit tests, such as the Hosmer–Lemeshow test, are used to evaluate calibration, or if for models predicting survival outcomes performance measures accounting for censoring are not used, or if classification measures (like sensitivity, specificity, or predictive values) were presented using predicted probability thresholds derived from the data set at hand.  No information: Either calibration or discrimination are not reported, or no information is provided as to whether appropriate performance measures for survival outcomes are used (e.g., references to relevant literature or specific mention of methods, such as using Kaplan–Meier estimates), or no information on thresholds for estimating classification measures is given. | Yes/  probably yes  🗹 | No/  probably no  □ | No  Information  □ |
| **4.8 Were model overfitting and optimism in model performance accounted for?†**  Yes/probably yes: If internal validation techniques, such as bootstrapping and cross-validation including all model development procedures, have been used to account for any optimism in model fitting, and subsequent adjustment of the model performance estimates have been applied.  No/probably no: If no internal validation has been performed, or if internal validation consists only of a single random split-sample of participant data, or if the bootstrapping or cross-validation did not include all model development procedures including any variable selection.  No information: No information is provided on whether internal validation techniques, including all model development procedures, have been applied. | Yes/  probably yes  🗹 | No/  probably no  □ | No  Information  □ |
| **4.9 Do predictors and their assigned weights in the final model correspond to the results from the reported multivariable analysis?†**  Yes/probably yes: If the predictors and regression coefficients in the final model correspond to reported results from multivariable analysis.  No/probably no: If the predictors and regression coefficients in the final model do not correspond to reported results from multivariable analysis.  No information: If it is unclear whether the regression coefficients in the final model correspond to reported results from multivariable analysis. | Yes/  probably yes  □ | No/  probably no  □ | No  Information  🗹 |
| **Risk of bias introduced by the analysis**  Low risk of bias: If the answer to all signaling questions is “Yes” or “Probably yes,” then risk of bias can be considered low.  If ≥1 of the answers is “No” or “Probably no,” the judgment could still be low risk of bias, but specific reasons should be provided why the risk of bias can be considered low.  High risk of bias: If the answer to any of the signaling questions is “No” or “Probably no,” there is a potential for bias.  Unclear risk of bias: If relevant information about the analysis is missing for some of the signaling questions but none of the signaling question answers is judged to put the analysis at high risk of bias. | Low risk of bias  □ | High risk of bias  🗹 | Unclear risk of bias  □ |

EPV = events per variable.

* For EPVs between 10 and 20, the item should be rated as either probably yes or probably no, depending on the outcome frequency, overall model performance, and distribution of the predictors in the model.

† Development only.

| **Overall Assessment of Risk of Bias** | |
| --- | --- |
| **Rating** | **Criteria** |
| Low risk of bias □ | If all domains were rated low risk of bias.  If a prediction model was developed without any external validation, and it was rated as low risk of bias for all domains, consider downgrading to high risk of bias. Such a model evaluation can only be considered as low risk of bias, if the development was based on a very large data set and included some form of internal validation. |
| High risk of bias 🗹 | If ≥1 domain is judged to be at high risk of bias. |
| Unclear risk of bias □ | If an unclear risk of bias was noted in ≥1 domain and it was low risk for all other domains. |
