# Supplementary figures and images for: Development of Two Diagnostic Prediction Models for Leptomeningeal Metastasis in Patients With Solid Tumors
Source: Front Neurol. 2022 May 23;13:899153. doi: 10.3389/fneur.2022.899153 (PMC9168081; doi:10.3389/fneur.2022.899153)

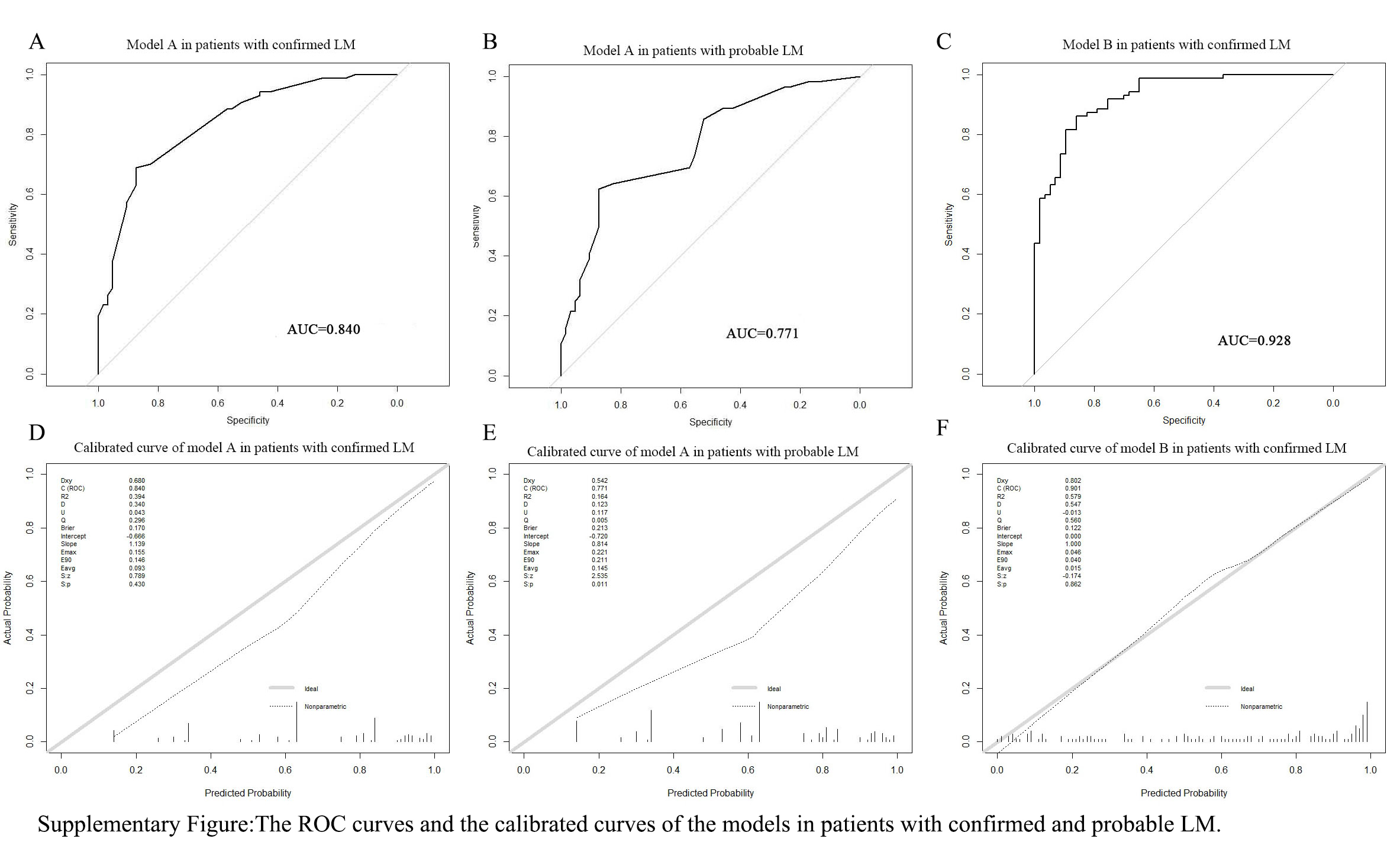

Supplement: Supplementary file 3 [file Image_1.JPEG]
